# pH-responsive drug delivery system based on hollow silicon dioxide micropillars coated with polyelectrolyte multilayers

**DOI:** 10.1186/1556-276X-9-411

**Published:** 2014-08-21

**Authors:** María Alba, Pilar Formentín, Josep Ferré-Borrull, Josep Pallarès, Lluís F Marsal

**Affiliations:** 1 Departament d'Enginyeria Electrònica, Elèctrica i Automàtica, Universitat Rovira i Virgili, Avda. Països Catalans 26, Tarragona 43007, Spain

**Keywords:** Hollow micropillars, Porous silicon, Drug delivery, pH-responsive, Controlled release, Doxorubicin

## Abstract

We report on the fabrication of polyelectrolyte multilayer-coated hollow silicon dioxide micropillars as pH-responsive drug delivery systems. Silicon dioxide micropillars are based on macroporous silicon formed by electrochemical etching. Due to their hollow core capable of being loaded with chemically active agents, silicon dioxide micropillars provide additional function such as drug delivery system. The polyelectrolyte multilayer was assembled by the layer-by-layer technique based on the alternative deposition of cationic and anionic polyelectrolytes. The polyelectrolyte pair poly(allylamine hydrochloride) and sodium poly(styrene sulfonate) exhibited pH-responsive properties for the loading and release of a positively charged drug doxorubicin. The drug release rate was observed to be higher at pH 5.2 compared to that at pH 7.4. Furthermore, we assessed the effect of the number of polyelectrolyte bilayers on the drug release loading and release rate. Thus, this hybrid composite could be potentially applicable as a pH-controlled system for localized drug release.

## Background

Micro- and nanoporous structures based on the electrochemical etching of porous silicon have attracted much attention in medical and biotechnological applications owing to their biodegradability, nontoxicity and versatile physico-chemical properties, including surface functionality, size and porosity [1-5]. The combination of electrochemical etching and microfabricaton techniques have also enabled the fabrication of neatly defined and monodispersed structures with a precise control on particle dimensions and shape, which can be critical for eliminating variability, improving pharmacokinetics and adapting microscale features in several bioapplications [6-9]. Particularly, hollow silicon dioxide (SiO_2_) micropillars exhibit remarkable advantages such as high chemical and mechanical stability, tunable size and functional modifiable surface [10,11]. These 3D structures are obtained from silicon macropores produced on lithographically pre-patterned silicon wafers [12]. The conformal growth of thermal SiO_2_ opens the way for the formation of inverted structures [10,13]. The hollow volume of micropillars can be loaded with active species, such as drugs, bioactive agents, enzymes and antibiotics. Furthermore, a differential inner/outer functionalization can activate the external surface in order to facilitate the interaction with species grafted on the external side [11].

Compared to conventional form of dosage, micro- and nanomaterial-based drug delivery systems have many advantages, such as reduced release rate, minimized harmful side effects and improved therapeutic efficiency [7,14,15]. However, the premature release of active species from the cargo-loaded micropillars can represent a drawback. Hence, a triggered and prolonged release of guest molecules upon specific stimuli may be desired. This stimulus for the drug delivery system can be induced by physical [16], chemical [17] or biogenic signals [18]. In this context, polyelectrolyte multilayer (PEM) has been widely explored to create coatings on the surface of a number of inorganic structures for the controlled delivery of drugs [19-23]. The PEM assembly is based on the layer-by-layer (LbL) approach which involves alternative adsorption of oppositely charged polyelectrolytes to create multilayer architectures in a conformal manner [24-26]. By the incorporation of appropriate responsive polyelectrolytes, the PEM can allow the controlled release of active agents on the basis of stimuli such as pH [27], temperature [28] or ionic strength [29]. Particularly, pH-sensitive systems are of great interest in drug delivery due to the variations in pH that the human body exhibits. For instance, the gastrointestinal tract exhibits pH ranging from acidic in the stomach (pH 2) to basic in the intestine (pH 5 to 8). And compared to healthy tissues and the bloodstream (pH 7.4), most cancer and wound tissues constitute an acidic environment (pH 7.2 to 5.4) [30]. pH-responsive PEM films contain ionizable groups which exhibit volume changes in response to variations in pH and facilitate drug delivery control [31].

The polyelectrolyte pair comprising poly(allylamine hydrochloride) (PAH) and sodium poly(styrene sulfonate) (PSS) has been extensively investigated for drug delivery applications due to their remarkable sensitivity to pH and improved biocompatibility [20,32]. The deposition of the first layer of cationic polyelectrolyte PAH on the internal sidewalls of hollow micropillars is favoured by the negative charge of the SiO_2_ surface above the isoelectric point (pH 2 to 3) [33]. Then, the anionic PSS is deposited onto PAH by electrostatic attraction. Furthermore, to facilitate the infiltration of the polyelectrolytes inside the pores and obtain a uniform surface coating without pore blockage, a multivalent salt such as CaCl_2_ can be added to the aqueous polyelectrolyte solution. The presence of multivalent salts causes a much stronger shrinking of the polyelectrolyte chain owing to a higher attraction between charged monomers along the chain [34,35]. The infiltration of the drug inside the polyelectrolyte multilayer can also be assisted by electrostatic attractive forces. The negative charge of the most external PSS layer gives extra electrostatic attraction to positively charged drugs, such as doxorubicin hydrochloride (DOX). DOX is a chemotherapeutic agent widely used in the treatment of a number of tumours, such as breast, lung or ovarian cancers [36,37]. Its inherent fluorescence gives DOX an additional imaging capability which makes it a remarkable theranostic agent [14,38-40].

Herein, we present the combination of SiO_2_ micropillars with PEM coating as an approach to develop new functional materials for sustained release of drug molecules. The hollow micropillars are used as reservoirs for doxorubicin and the PAH/PSS coating as a pH-responsive switch. The polyelectrolyte multilayer on the interior surface prevents the premature release of the drug and enables an enhanced use of the hollow volume by increasing the loading capacity. The effect of the number of PAH/PSS layers in the drug loading and release is also investigated.

## Methods

### Materials

Hydrofluoric acid (HF, 40%), N,N-dymethylformamide (DMF), buffered hydrofluoric acid (BHF) and tetramethylammonium hydroxide (TMAH, 25%), PAH (M_w_ 58,000) and PSS (M_w_ 70,000) were purchased from Sigma-Aldrich (St. Louis, MO, USA). Acetate buffer (ABS) pH 5.2 and phosphate buffer (PBS) pH 7.4 solutions were also obtained from Sigma-Aldrich. Doxorubicin hydrochloride was obtained from the European Pharmacopoeia (Strasbourg, France). All other chemicals used in the experiments were obtained from commercial sources as analytical reagents without further purification. Milli-Q water (Millipore, Billerica, MA, USA) with a resistivity of 18.2 MΩ cm was used throughout the study. Boron-doped (*p*-type) silicon wafers (1 0 0) and resistivity 10 to 20 Ω cm were supplied by Si-Mat (Kaufering, Germany).

### Fabrication of SiO_2_ micropillars

**Figure 1:**
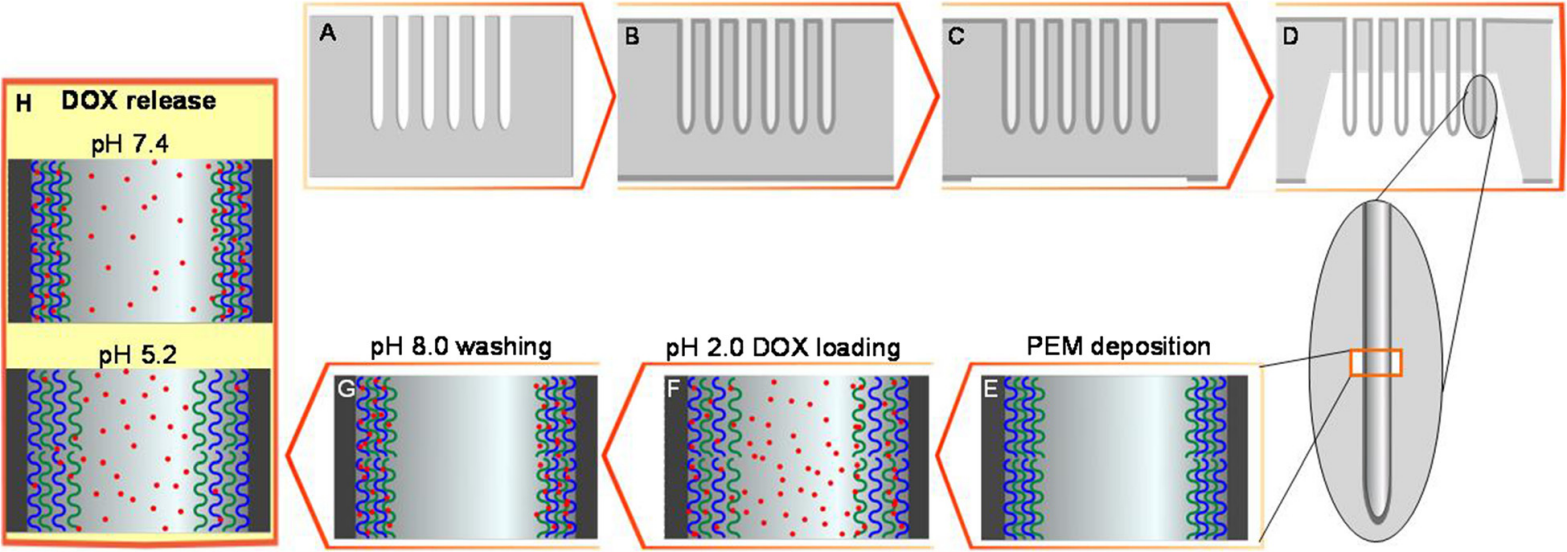
**Schematic of the process for the micropillar fabrication, PEM coating and DOX loading and release. (A)** Macropore formation by electrochemical etching; **(B)** SiO_2_ growth by thermal oxidation; **(C)** backside oxide removal; **(D)** backside silicon removal by TMAH etching and micropillar releasing; **(E)** polyelectrolyte pair PAH/PSS alternative deposition on the internal sidewalls of SiO_2_ micropillars via the LbL method to construct a pH-responsive drug delivery system; **(F)** DOX loading in the swollen PEM film at pH 2.0; **(G)** DOX confinement due to the PEM layer contraction at pH 8.0; and **(H)** DOX release in different media at pH 7.4 and 5.2.

SiO_2_ micropillars were fabricated from macroporous silicon produced by electrochemical etching in *p*-type silicon wafers following the process described elsewhere [10-12]. In order to obtain regular pore arrays, the Si wafer was pre-patterned with a 3-μm lattice using a direct-write lithography system (DWL 66FS, Heidelberg Instruments Gmbh, Heidelberg, Germany). Macropores were formed under galvanostatic conditions (5 mA cm^−2^) in a solution of 1:10 (*v*/*v*) HF (40%wt) to DMF (A in Figure 1). Following, the sample was oxidized at 1,000°C for 1.5 h in air (B in Figure 1). Then, the backside of the wafer was patterned to open windows where the oxide layer was removed by BHF etching (C in Figure 1). Finally, the silicon bulk was anisotropically etched in TMAH (12%, 85°C). As a result, the SiO_2_ micropillars appear protruding out of the backside of the silicon wafer (D in Figure 1).

### Polyelectrolyte multilayer coating

PAH/PSS multilayer coating was deposited by alternately exposing the internal side of the micropillar sample to solutions of PAH and PSS (1 mg mL^−1^ in CaCl_2_ 0.5 M) for 20 min each in an ultrasonic bath (E in Figure 1). After the deposition of each polyelectrolyte, the sample was thoroughly washed twice in Milli-Q water for 5 min each. This sequence was repeated until obtaining the desired number (4, 8 or 12) of PAH/PSS bilayers.

### Characterization instruments

The morphology and structure of the macroporous silicon and subsequent silicon dioxide micropillars were characterized by scanning electron microscopy (SEM) using a FEI Quanta 600 environmental scanning electron microscope (FEI, Hillsboro, OR, USA) operating at an accelerating voltage between 15 and 25 kV. The micropillars were also morphologically characterized by transmission electron microscopy (TEM) using a JEOL 1011 (JEOL Ltd., Akishima-shi, Japan) operating in dark-field mode at 80 kV. Confocal laser scanning microscopy images were taken using a Nikon Eclipse TE2000-E inverted microscope, equipped with a C1 laser confocal system (EZ-C1 software, Nikon, Tokyo, Japan). A 488-nm helium-neon laser was used as excitation source for DOX-loaded micropillars. The emission was collected through a 590 ± 30 bandpass emission filter (red channel). All fluorescence images were captured using a 5-megapixel CCD. The concentrations of DOX were determined using a spectrofluorometer (PTI Quantamaster 40, Photon Technologies International, Edison, NJ, USA) at an exciting wavelength of 480 nm.

### DOX loading and pH-responsive drug release

Doxorubicin was loaded inside the PEM-coated micropillar, as well as in bare SiO_2_ samples. To perform the drug loading, the micropillar samples were exposed to a solution of DOX 1 mg mL^−1^, adjusted to pH 2.0 with HCl 1 M, for 20 h in the dark (F in Figure 1). Then, DOX solution was adjusted to pH 8.0 with NaOH 0.1 M and further stirred for 2 h (G in Figure 1). The drug-loaded samples were washed three times in water at pH 8 for 10 min each. The amount of released DOX in solutions of pH 7.4 (phosphate buffer) and 5.2 (acetate buffer) was monitored over time (up to 24 h) at an exciting wavelength of 480 nm (H in Figure 1).

## Results and discussion

**Figure 2:**
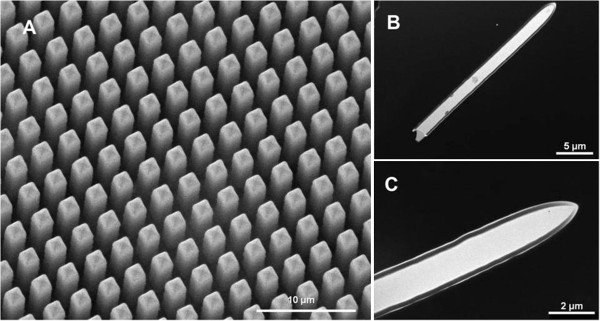
**Microscopy characterization of the SiO**_**2 **_**micropillars.** SEM image of released micropillars with a diameter of 1.8 μm **(A)**, and dark-field TEM images of a detached micropillar with a length of 26 μm **(B)** and a detail of the uniform SiO_2_ wall and hollow structure on the micropillar tip **(C)**.

Figure 2A shows a SEM image of SiO_2_ micropillars with a diameter of 1.8 μm, protruding out of the backside of the Si wafer. The micropillar arrays retain the same arrangement and dimensions as the preceding macropores. Their nucleation points were defined by lithography-assisted indentation so that the distance between neighbouring micropillars is set to 3 μm. The thermal oxide grows in a conformal manner which preserves the ordering, morphology and uniformity of those initial macropores. The micropillar hollow structure was further investigated by TEM. Figure 2B shows a cross-section-like dark field TEM image of a detached micropillar with a length of 26 μm and a regular wall thickness all along. A detail of the micropillar closed-end is presented in Figure 2C with a thermally grown SiO_2_ wall approximately 150 nm thick.

**Figure 3:**
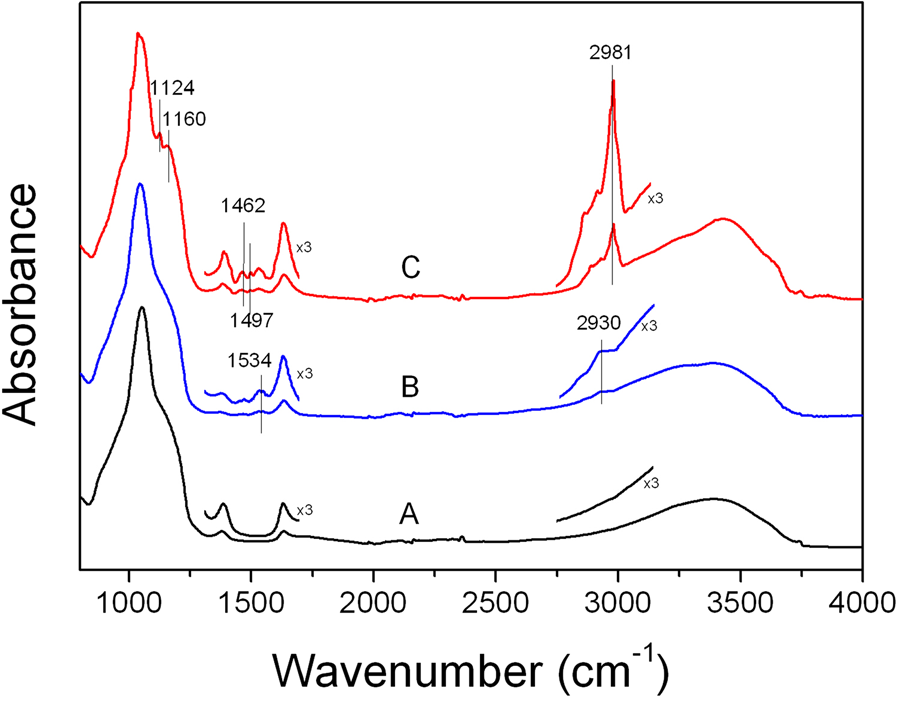
**FTIR-ATR characterization for polyelectrolyte coating.** FTIR-ATR spectra of (A) oxidized, (B) PAH-coated, and (C) PAH/PSS-coated macroporous silicon.

Fourier transform infrared-attenuated total reflection (FTIR-ATR) spectroscopy was employed to verify the electrostatic deposition of the polyelectrolytes on the micropillar sample. Bare SiO_2_ possesses a negative surface charge above the isoelectric point (pH 1.7 to 3.5) [41], which facilitates the cationic PAH adsorption. After PAH deposition, an absorption band appears at approximately 2,930 cm^−1^ related to the C-H_x_ stretching vibrations, although it is distorted by the broad νOH band. The band centred at approximately 1,534 cm^−1^ is attributed to the N-H bending modes in NH_3_^+^ (Figure 3, spectrum B). These findings prove successful adsorption of the PAH on the silicon oxide. The FTIR-ATR of the sample with a bilayer of PAH/PSS shows bands related to the C-C stretching modes of the aromatic ring in the PSS molecule at 1,497 and 1,462 cm^−1^ (Figure 3, spectrum C). The contribution of the alkyl CH_2_ symmetric stretching components from PSS incorporates to those of PAH in the 2,800 to 3,000 cm^−1^ region. However, a new intense band appears at 2,981 cm^−1^ which can be attributed to the C-H stretching in the PSS aromatic ring. The symmetric and asymmetric stretching regions of SO_3_^−^ overlap with the νSiO_x_ absorption between 900 and 1,250 cm^−1^. Nevertheless, at least two peaks can be discerned at 1,124 and 1,160 cm^−1^ corresponding to the SO_3_^−^ stretching vibrations [42,43]. These observations confirm the successful deposition of PAH and PSS polyelectrolytes on the silicon dioxide micropillars.

**Figure 4:**
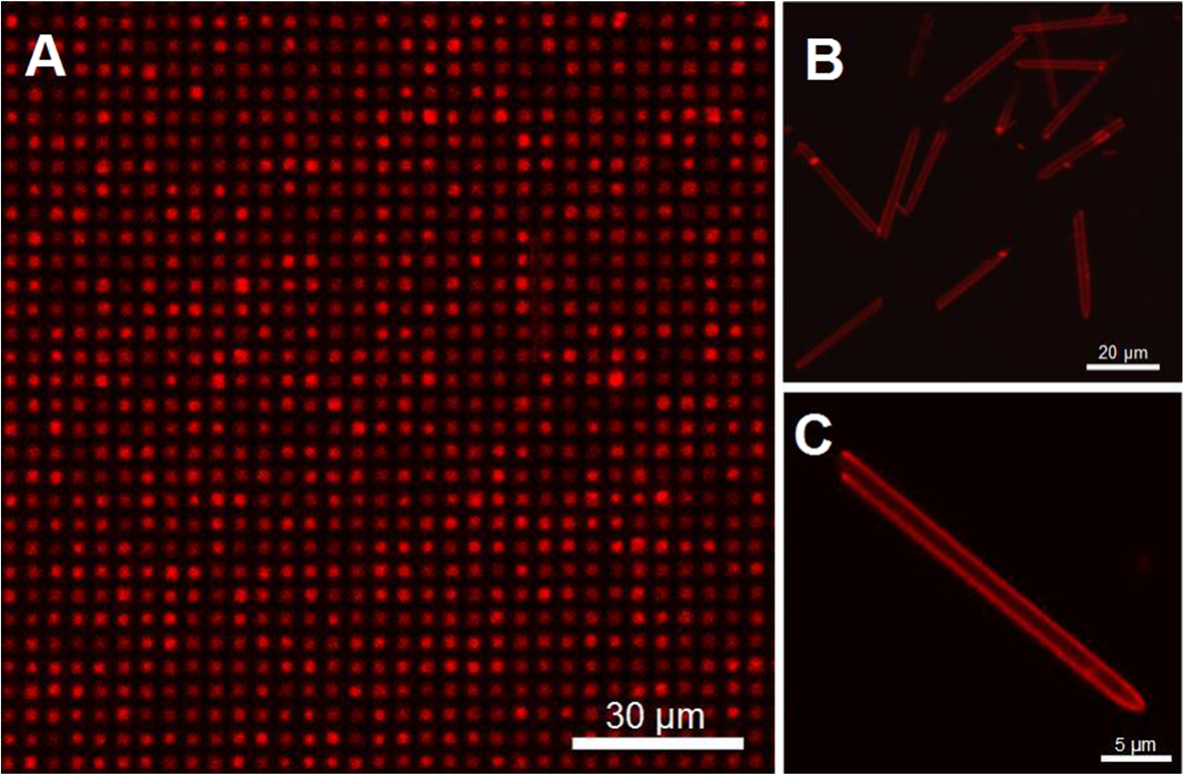
**Fluorescence confocal images of PEM-coated and DOX-loaded micropillars.** Fluorescence confocal micrograph of the micropillar arrays in top view after PEM coating (eight bilayers) and DOX loading **(A)**; detached hollow micropillars with uniform size distribution **(B)**; and single detached micropillar with PEM and DOX all along the walls **(C)**.

Confocal fluorescence microscopy was used to confirm drug adsorption into the polyelectrolyte multilayer, as well as to verify the PEM coating conformation inside the micropillars. Firstly, we imaged a top view of the micropillar arrays after coating with eight bilayers PAH/PSS and loading with DOX for 20 h at pH 2.0, then 2 h at pH 8.0 and thoroughly washed with deionized water (DIW) pH 8.0. At pH 2.0, the increased permeability of the PEM film facilitates the incorporation of DOX inside the PAH/PSS multilayers. Additionally, the negatively charged PSS outer layer promotes the electrostatic adsorption of the positively charged DOX. Then, the adjustment of pH at 8.0 causes the shrinkage of the PEM, and the drug molecule is trapped inside the film. The subsequent washing will remove any nontrapped DOX molecule. Figure 4A was collected exposing the micropillar arrays to a laser excitation of 488 nm and using a 590 ± 30-nm bandpass emission filter (red channel). Bright red dots appear in correspondence with the micropillar pattern, which confirms the pH-controlled adsorption of DOX in the PAH/PSS multilayer. In addition, PEM-coated and DOX-loaded micropillars were detached from the silicon substrate in order to analyse the conformation of the polyelectrolyte multilayer and, subsequently, the DOX adsorption. Figure 4B shows a number of micropillars with uniform size and shape, exhibiting bright red fluorescence originated from the loaded DOX. This observation indicates a successful deposition of the polyelectrolyte multilayer on the micropillar sidewalls, in which no pore blockage occurred during the LbL self-assembly. The use of a multivalent salt such as CaCl_2_ assists the formation of the polyelectrolyte layer inside the micropillars owing to a stronger polymer-chain contraction [34]. Figure 4C shows a closer detail of a single hollow micropillar with a homogeneous distribution of the DOX all along their wall, confirming the conformational PEM deposition along the micropillar walls.

**Figure 5:**
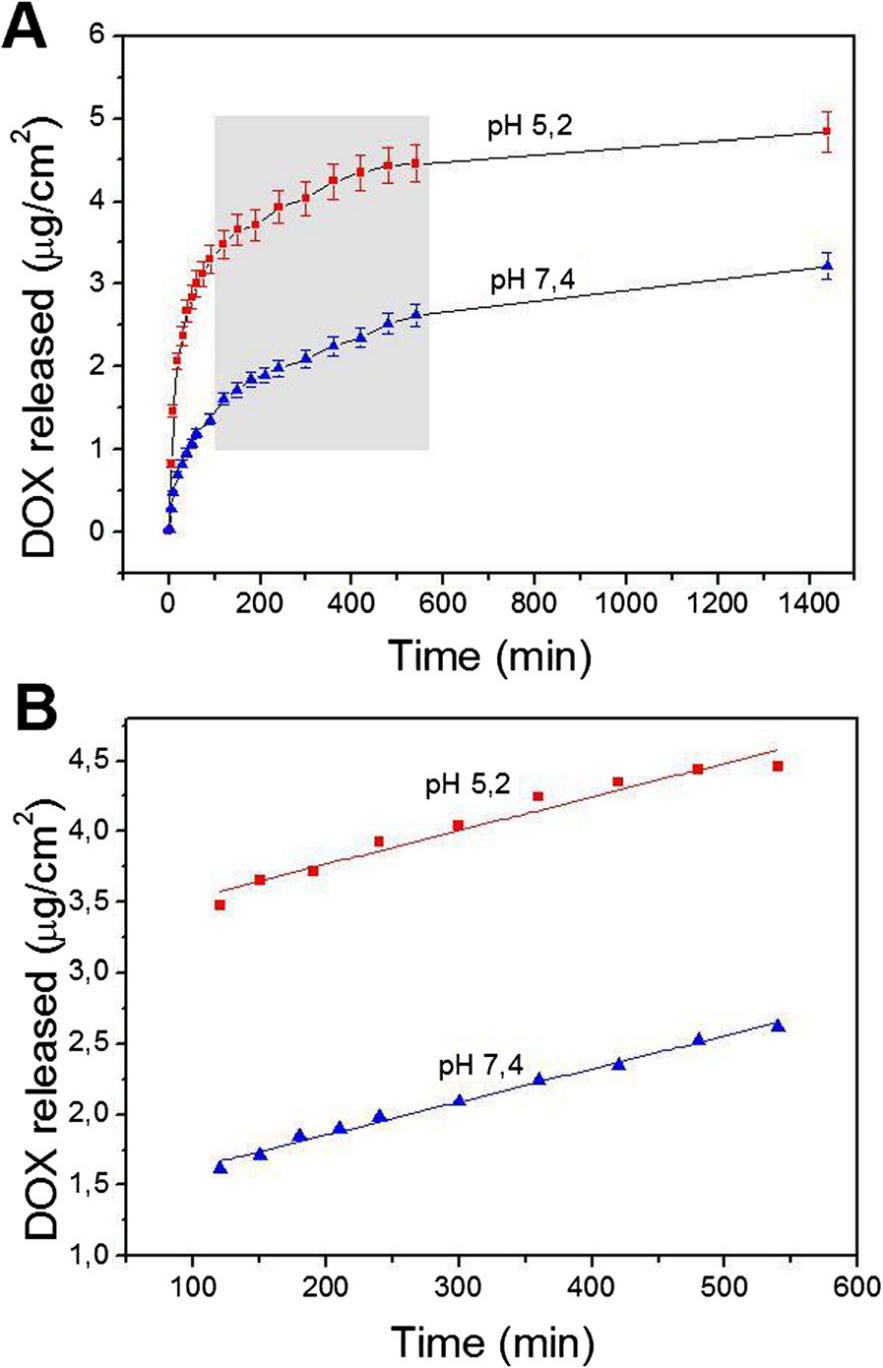
**Drug release profile for 24 h at pH 7.4 and 5.2. (A)** Time evolution of pH-responsive release of DOX from PEM-coated (eight bilayers) micropillars at pH 5.2 (red squares) and 7.4 (blue triangles); **(B)** zoomed-in plot and linear fitting of the DOX release in the region between 120 and 540 min.

After the DOX loading, the micropillars were exposed to two different pH media to assess the pH responsiveness. Once in contact with the aqueous medium, the PEM film swells to a certain extent, increasing its permeability and allowing the diffusion of the drug. After the DOX releasing from the PEM film, the molecule still remains inside the micropillar until it finally diffuses into the release medium through the micropillar opened-end. Figure 5A compares the release profile of DOX from the PEM-coated micropillars at pH 5.2 and 7.4 over a period of 24 h. The data indicates that the release at pH 5.2 is higher than that at pH 7.4 (4.8 and 3.2 μg cm^−2^ after 24 h, respectively). This demonstrates the release rate is pH-dependent and increases with the decrease of pH. The swelling mechanism of PAH/PSS films is mostly related to the variation in charge density of polyelectrolyte chains induced by a change in the media pH. PAH is a weak polyelectrolyte whose amino groups become charged when the pH decreases, causing an increase in the osmotic pressure. Subsequently, water molecules diffuse into the PEM and the multilayer swells. This phenomenon, together with the electrostatic repulsion between DOX and the PAH/PSS multilayer, facilitates the permeation of the drug [44]. Furthermore, the DOX discharge from the multilayer at pH 5.2 shows a considerable burst release within the first 90 min (71.3% of the total release after 24 h), which is mitigated by the deswelling effect on the PEM at pH 7.4 (46.97%). Considering absolute values, the DOX released after 60 min at pH 5.2 is nearly 2.5 times higher than that at pH 7.4 (3.3 and 1.3 μg cm^−2^, respectively). Then, the release rate slows and becomes rather constant from 120 min for both pH 5.2 and 7.4, lasting approximately 7 h (Figure 5B). At this point, the effect of the pH in the release rate is negligible, being 2.38 and 2.34 μg cm^−2^ min at pH 5.2 and 7.4, respectively.

**Figure 6:**
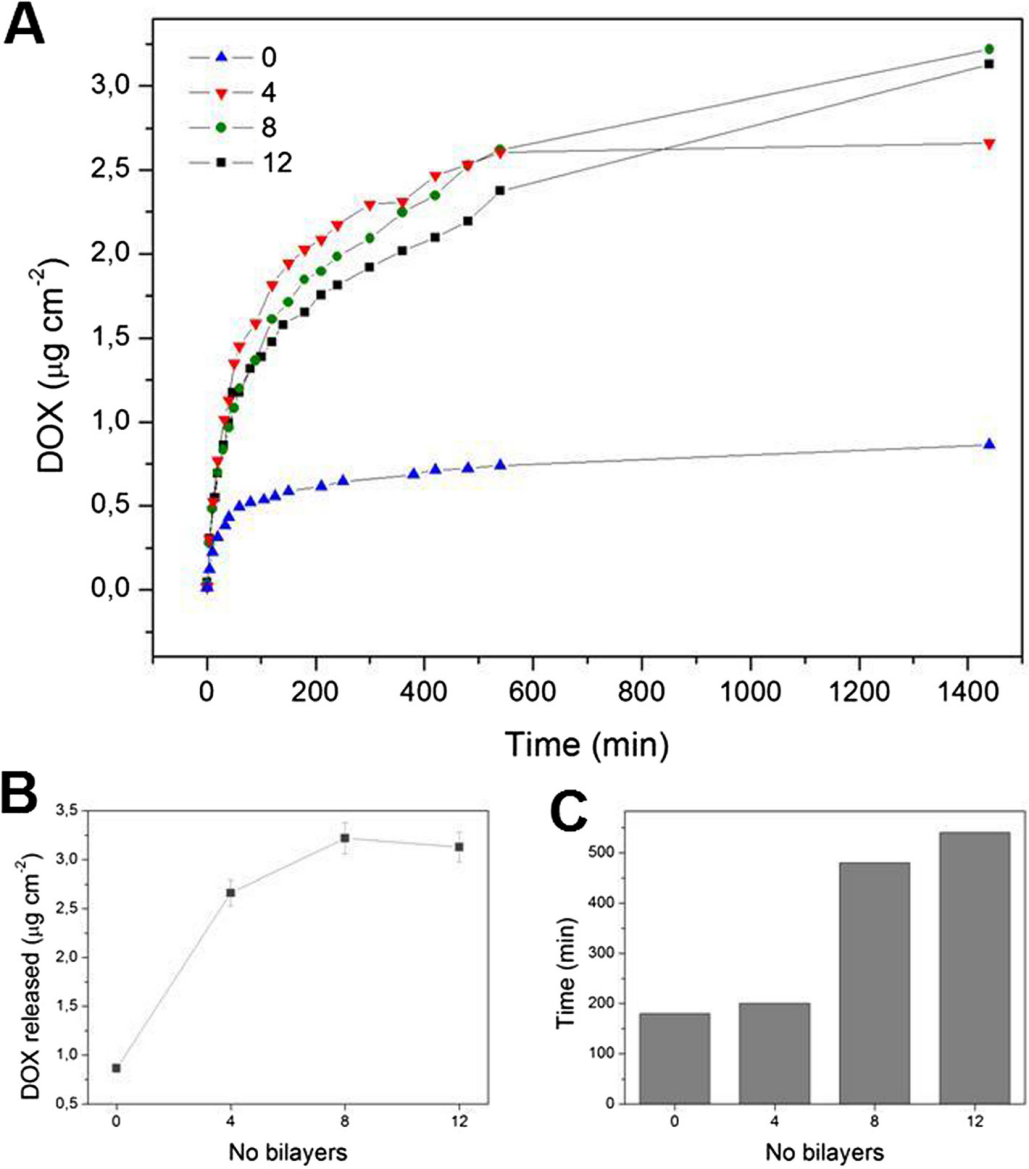
**Effect of the bilayer number in the DOX release. (A)** Release profiles of DOX from PEM coated with different layer numbers (pH 7.4); **(B)** DOX released after 24 h and **(C)** time to reach the 80% of the total release as a function of the number of layers.

The effect of the number of bilayers in DOX loading and release was also investigated at pH 7.4. Figure 6A revealed that the loading content and release rate of DOX was layer thickness-dependent. The drug loaded was observed to be significantly higher in the PEM-coated micropillars than in those without multilayers (Figure 6B). Thus, the amount of DOX released after 24 h at pH 7.4 was three times higher in samples with four PAH/PSS layers compared to samples without polyelectrolyte (2.66 and 0.86 μg cm^−2^, respectively). Although the deposition of PEM increases the loading capacity due to an enhanced electrostatic interaction and permeability of the PEM layer, it is worth noticing that positively charged DOX molecules can still be adsorbed onto the negatively charged SiO_2_ micropillar walls. When further increasing the number of bilayers, the abrupt increase in the amount of DOX loaded and released was not notably improved. The release rate was also affected by the number of layers. Figure 6C shows that the time to reach 80% of the total DOX release after 24 h (1,440 min) was delayed with the number of layers. For instance, it was found that this time was 200 and 480 min for samples with four and eight PAH/PSS layers, respectively. Thus, by adding more PEM bilayers, it is possible to significantly reduce the release rate and impede the initial burst release.

## Conclusions

In summary, an organic/inorganic hybrid drug delivery system was developed based on SiO_2_ hollow micropillars internally coated with multilayers of PAH/PSS by the LbL technique. Confocal fluorescence microscopy showed a uniform PEM coating and a successful loading of the model drug doxorubicin into the polyelectrolyte matrix. The interaction between polyelectrolyte multilayers and DOX molecules is significantly dependent on the pH for the loading and release of active agents. Thus, the release rate of DOX at pH 5.2 was found to be higher than that at pH 7.4. The effect of the number of PAH/PSS bilayers should be also considered in the drug loading. The DOX loaded was significantly higher in the PEM-coated micropillars than in those without polyelectrolytes. This system has great potential in applications of localized and targeted drug delivery.

## Abbreviations

DOX: doxorubicin hydrochloride; PAH: poly(allylamine hydrochloride); PSS: sodium poly(styrene sulfonate); PEM: polyelectrolyte multilayer; LbL: layer-by-layer; TEM: transmission electron microscopy; SEM: scanning electron microscopy; HF: hydrofluoric acid; DMF: N,N-dymethylformamide; BHF: buffered hydrofluoric acid; TMAH: tetramethylammonium hydroxide; ABS: acetate buffer; PBS: phosphate buffer.

## Competing interests

The authors declare that they have no competing interests.

## Authors' contributions

The experiments presented in this work were designed by MA and LFM. The complete process of the SiO_2_ micropillar fabrication was carried out by MA and PF. MA characterized by SEM, TEM and confocal microscopy. PF assisted MA during the laboratory tasks. MA, PF, JFB, JP and LFM analysed and discussed the results obtained from the experiments. MA wrote the manuscript, and the last version of this was revised by all the authors (MA, PF, JFB, JP and LFM). All authors read and approved the final manuscript.
